# Nimotuzumab Induces NK Cell Activation, Cytotoxicity, Dendritic Cell Maturation and Expansion of EGFR-Specific T Cells in Head and Neck Cancer Patients

**DOI:** 10.3389/fphar.2017.00382

**Published:** 2017-06-19

**Authors:** Zaima Mazorra, Anabel Lavastida, Fernando Concha-Benavente, Anet Valdés, Raghvendra M. Srivastava, Tatiana M. García-Bates, Esperanza Hechavarría, Zuyen González, Amnely González, Martha Lugiollo, Iván Cuevas, Carlos Frómeta, Braulio F. Mestre, Maria C. Barroso, Tania Crombet, Robert L. Ferris

**Affiliations:** ^1^Department of Clinical Immunology, Clinical Direction, Center of Molecular ImmunologyHavana, Cuba; ^2^Department of Immunology, University of Pittsburgh, PittsburghPA, United States; ^3^Department of Otolaryngology, University of Pittsburgh, PittsburghPA, United States; ^4^Department of Infectious Diseases and Microbiology, Graduate School of Public Health, University of Pittsburgh, PittsburghPA, United States; ^5^National Institute of Oncology and RadiobiologyHavana, Cuba; ^6^Clinical Direction, Center of Molecular ImmunologyHavana, Cuba; ^7^Cancer Immunology Program, University of Pittsburgh Cancer Institute, PittsburghPA, United States

**Keywords:** human epidermal growth factor receptor, head and neck cancer, monoclonal antibodies, natural killer cells, T cells

## Abstract

Survival benefit and long-term duration of clinical response have been seen using the epidermal growth factor receptor (EGFR)-targeted monoclonal antibody (mAb) nimotuzumab. Blocking EGFR signaling may not be the only mechanism of action underlying its efficacy. As an IgG1 isotype mAb, nimotuzumab’s capacity of killing tumor cells by antibody dependent cellular cytotoxicity (ADCC) and to induce an immune response in cancer patients have not been studied. ADCC-induced by nimotuzumab was determined using a ^51^Cr release assay. The *in vitro* effect of nimotuzumab on natural killer (NK) cell activation and dendritic cell (DC) maturation and the *in vivo* frequency of circulating regulatory T cells (Tregs) and NK cells were assessed by flow cytometry. Cytokine levels in supernatants were determined by ELISA. ELISpot was carried out to quantify EGFR-specific T cells in nimotuzumab-treated head and neck cancer (HNSCC) patients. Nimotuzumab was able to kill EGFR+ tumor cells by NK cell-mediated ADCC. Nimotuzumab-activated NK cells promoted DC maturation and EGFR-specific CD8+ T cell priming. Interestingly, nimotuzumab led to upregulation of some immune checkpoint molecules on NK cells (TIM-3) and DC (PD-L1), to a lower extent than another EGFR mAb, cetuximab. Furthermore, circulating EGFR-specific T cells were identified in nimotuzumab-treated HNSCC patients. Notably, nimotuzumab combined with cisplatin-based chemotherapy and radiation increased the frequency of peripheral CD4+CD39+FOXP3+Tregs which otherwise were decreased to baseline values when nimotuzumab was used as monotherapy. The frequency of circulating NK cells remained constant during treatment. Nimotuzumab-induced, NK cell-mediated DC priming led to induction of anti-EGFR specific T cells in HNSCC patients. The association between EGFR-specific T cells and patient clinical benefit with nimotuzumab treatment should be investigated.

## Introduction

Tumor antigen (TA)-targeted monoclonal antibodies (mAbs) have demonstrated clinical success against different types of tumors but only in a limited proportion of patients. Understanding the mechanisms of action of these therapies would optimize the selection of patients that are most likely to benefit. FDA-approved mAbs such as rituximab (anti-CD20), trastuzumab (anti-HER2), and cetuximab (anti-HER1/anti-EGFR) used in lymphoma, breast cancer, head and neck, and colorectal carcinomas, respectively, not only block tumor cell signaling but also induce innate and adaptive antitumor immunity (Ferris et al., 2010). For instance, the anti-EGFR mAb cetuximab induced TA specific CD8+ T cell priming, via natural killer (NK) cell-induced dendritic cell (DC) maturation, which lead to TA spreading and Th1 cytokine release (Lee et al., 2011). Likewise, elevated circulating EGFR-specific CD8+ T cells were found in cetuximab-treated patients with head and neck cancer (HNSCC) as compared with cetuximab-naïve HNSCC patients (Srivastava et al., 2013). Despite the induction of TA specific immune response by cetuximab, treatment with this mAb also increases the frequency of circulating and intratumoral CTLA-4+ Foxp3+Tregs in HNSCC patients. These Tregs impair the NK-dependent ADCC by cetuximab using TGF-β1 secretion. Interestingly, the elevated frequency of Tregs was associated with poor clinical response to monotherapy with cetuximab (Jie et al., 2015).

Nimotuzumab is an IgG1 humanized mAb directed against the extracellular domain of the EGFR blocking the binding to its ligands (Mateo et al., 1997). Nimotuzumab has lower affinity for EGFR than cetuximab, since the dissociation constant (*K*_D_) for nimotuzumab is 2.1 × 10^-8^ mol/L (Talavera et al., 2009) and for cetuximab is 2.3 × 10^-9^ mol/L (Li et al., 2005). It has been speculated that nimotuzumab is safer than cetuximab since its toxic dose is higher than its clinical dose (Garrido et al., 2011). This could partially be justified by the capacity of this mAb to mainly direct at the tumor, in which the EGFR expression is higher than in normal epithelial cells. In preclinical studies, it was demonstrated that nimotuzumab is a strong antitumor drug both for *in vitro* and for *in vivo* setting by combining an antiproliferative, antiangiogenic and proapoptotic effect upon tumors cells that overexpress the EGFR (Crombet-Ramos et al., 2002).

In the clinical setting, nimotuzumab has demonstrated clinical efficacy in various epithelial tumors (Ramakrishnan et al., 2009; Reddy et al., 2014). Based on those results, it has achieved several approvals in Cuba including nasopharyngeal tumors, advanced head and neck carcinoma, esophageal cancer, adult and children brain tumors and more recently pancreatic cancer (Strumberg et al., 2012). The antibody also was approved in 28 other countries for treatment of some or all the above-mentioned tumors.

Overexpression of the EGFR is a hallmark of HNSCC (Cohen, 2006). In several phase II clinical trials, conducted in locoregionally advanced HNSCC the combination of nimotuzumab with radiotherapy (RT) or chemo-radiotherapy (CRT) significantly improved the overall survival (OS) and objective response in comparison with the conventional therapy alone (Reddy et al., 2014). In addition, a significant relationship between EGFR expression and OS in patients who received nimotuzumab plus CRT or RT as well as a direct correlation between EGFR overexpression and OS has been found (Basavaraj et al., 2010).

The increased survival and long-term duration of response seen in many patients after short treatment with nimotuzumab (Bode et al., 2012; Reddy et al., 2014), suggest that blocking EGFR signaling and inhibiting tumor cell proliferation might not be the only mechanisms of action underlying the efficacy of this antibody. Indeed, nimotuzumab’s capacity of killing tumor cells by ADCC, potentially inducing an immune response has been speculated, however, not characterized yet. Based on the findings of cetuximab and the long-term clinical responses seen with nimotuzumab, we investigated new potential mechanisms of action of this antibody that could explain its prolonged efficacy. Our study presents for the first time that nimotuzumab was able to kill EGFR+ tumor cells by NK cell-mediated ADCC. As previously reported for cetuximab, nimotuzumab also induces NK-DC cross-talk, which promotes DC maturation and EGFR-specific CD8+ T-cell priming *in vitro*. Interestingly, nimotuzumab induces the upregulation of some regulatory molecules such as TIM3 on NK cells and PD-L1 on DCs but at a lower level than cetuximab, under the same experimental conditions. Furthermore, EGFR-specific T cells were identified in nimotuzumab-treated HNSCC patients. Interestingly, the frequency of circulating Tregs significantly increased with the treatment with nimotuzumab combined with cisplatin-based chemotherapy and radiation. Nevertheless, 9 months after maintenance treatment with nimotuzumab as monotherapy, Tregs significantly decreased back to baseline values. NK cell frequency did not change during the treatment period.

## Materials and Methods

### Tumor Cell Lines

The HNSCC cell lines HLA-A2-EGFR+ PCI-15B and JHU-029 (Disis et al., 2009; Lopez-Albaitero et al., 2009a; Andrade Filho et al., 2010), were grown in Iscove’s modified Dulbecco’s medium (IMDM; Sigma) supplemented with 10% FBS (Cellgro), 2% L-glutamine, and 1% penicillin/streptomycin (Invitrogen) at 37°C in a 5% CO_2_, 95% humidity. Adherent tumor cells were detached by warm Trypsin–EDTA (0.25%) solution (Invitrogen).

### Patients and Treatment

Patients with histologically documented advanced (unresectable) locoregional HNSCC who were candidates for concurrent CRT were recruited in a physician-led clinical trial, registered with Cuban National Clinical Trials Registry (Trial ID: RPCEC00000241). Accessible via http://www.rpcec.sld.cu/trials/RPCEC00000219-En.

This was a single-center clinical trial in which 35 patients were recruited (**Table 1**). The study protocol was conducted in accordance to the principles of the Declaration of Helsinki and Good Clinical Practices guidelines and under the Investigational New Drug application authorized by the Cuban Regulatory Agency (CECMED). All patients provided written informed consent. Inclusion criteria included measurable lesions, age ≥ 18 years, ECOG performance status ≤ 2, life expectancy greater than 6 months and normal functioning of organs and bone marrow defined by absolute neutrophil count ≥ 1.5 × 10^9^/L, platelet count ≥ 100 × 10^9^/L, serum creatinine level ≤ the upper limit of normal and ALAT (alanine aminotransferase) and ASAT (alanine aspartate transaminase) level less than 2.5 times the upper normal limit. Main exclusion criteria were: prior radiotherapy or chemotherapy, concurrent active cancer, any uncontrolled intercurrent illness and pregnancy or lactation. All patients signed the informed consent. The protocol was approved by the Institutional Review Board of the National Institute of Oncology and Radiobiology. All patients received eight weekly infusions of nimotuzumab at 200 mg in combination with CRT during the induction phase. In the maintenance phase, patients received nimotuzumab at 200 mg every 21 days for at least 12 months. The primary endpoint of the trial was to study the immunological response induced by nimotuzumab and changes in frequency of lymphocyte populations. Therefore, blood samples for determining NK and Tregs cells frequency were collected prior to the first dose of nimotuzumab, after the induction phase (3 months) and at the end of the study (12 months). Peripheral blood mononuclear cells (PBMC) for IFNγ ELISpot assay were freshly collected from protocol patients who had been treated with nimotuzumab for at least 1 year (nine patients) and from other eight patients (five HNSCC, three other tumor localizations) which are receiving nimotuzumab for a prolonged time (2–8 years) (see **Table 1**). A control cohort of nine patients treated only with CRT was included. These subjects were gender and age-matched with the nimotuzumab patients. Blood samples were drawn at the same period after completing CRT.

**Table 1:** Demographics of the nimotuzumab-treated patients in this study.

Regimen	No. of Patients	Tumor site(No. of patients)	Mean age	Male	Female
RPCEC00000241	35	OC (1)	62.5	31	4
		OP (31)			
		L (1)			
		P (1)			
		NP (1)			
Compassionate use	8	AC (3)	53.7	6	2
		M (2)			
		Other (3)			

*Tumor site abbreviation: OC, oral cavity; OP, oropharynx; L, larynx; P, pharynx; NP, nasopharynx; AC, tonsil carcinoma; M, mesopharynx: Other: 1 uterine adenocarcinoma, 1 colon adenocarcinoma, 1 hypernephroma.*

### Processing of PBMC and Cell Isolation

Blood from patients with HNSCC included in the clinical trial was collected. PBMC were purified by Ficoll-Paque PLUS centrifugation (Amersham Biosciences) and used fresh for cell isolation and ELISpot assay or stored frozen. DCs were generated as described previously (Lopez-Albaitero et al., 2009b). CD14+ monocytes, NK cells, and CD8+ T cells were purified using EasySep kits (Stem cell technologies) and purity was more than 95% (Lopez-Albaitero et al., 2009a).

### Cellular Cytotoxicity Assay

Cytotoxicity was determined using a ^51^Cr release assay. Briefly, EGFR+ HLA-A2-HNSCC cell line JHU029 was used as target cells. Cells were incubated in 100 μL of media with 25 μCi of Na251CrO4 (PerkinElmer, Boston, MA, United States) for 60 min at 37°C and, then re-suspended in RPMI 1640 medium supplemented with 25 mM HEPES. Cells were thoroughly washed and plated at various effector: target (E:T) ratios in 96-well plates. Cetuximab, nimotuzumab, panitumumab or human IgG1 or IgG2 was added (10 μg/mL), then freshly purified NK cells were added at the specified E:T ratios. Plates were incubated for 4 h at 37°C in a 5% CO_2_ atmosphere. Controls for spontaneous (cells only) and maximal lysis (cells treated with 1% Triton-X) and specificity mAb control (human IgG1and IgG2 isotype) were included. Each reaction was done in triplicate and repeated three times. The supernatants were collected and analyzed with a PerkinElmer 96-well plate gamma counter. Results were normalized with the following formula: lysis = (experimental lysis – spontaneous lysis)/(experimental lysis – maximal lysis) × 100. Results are representative of two different donors and were plotted in bar graphs for interpretation.

### *In Vitro* Stimulation of EGFR-Specific CD8+ T Cells

Autologous NK and DC from HLA-A2+ donor were incubated with irradiated EGFR+ HNSCC tumor cells (PCI-15B) in the presence or not of anti-EGFR mAb (10 μg/mL). After 48 h NK primed-DCs were incubated with autologous negatively isolated CD8+ T cells for 7 days at 37°C with rhIL-2 (20 U/mL) and rhIL-7 (5 ng/mL). On day 7, lymphocytes were re-stimulated with autologous DC previously primed with NK: PCI-15B (1:1:1 ratio) in the presence or not of anti-EGFR mAbs. Culture medium (IMDM) was supplemented with IL-2 (20 U/mL) and IL-7 (5 ng/mL) as cells needed. After 7 days, CD8+ T cells were harvested and stained with CD3, CD8, zombie aqua and HLA-A2+EGFR_853-861_ tetramer and analyzed by flow cytometry. Events were gated for viable (zombie aqua_neg_) lymphocytes, excluding doublets, that were CD3+CD8+ and analyzed the percentage of CD8+ T cells specific to HLA-A2+EGFR_853-861_ tetramer. HLA-A2 HIV peptide tetramer was used as negative control.

### Enzyme-Linked Immunosorbent Spot (ELISpot) Assay

EGFR-specific T cells secreting IFN-γ was assessed by standard IFN-γ ELISPOT kit (Mabtech AB). Briefly, PBMC were immediately isolated after blood collection by gradient centrifugation (Ficoll-Paque PLUS, Amersham Biosciences). After washing, PBMC was re-suspended in IMDM medium supplemented with 10% human serum AB and seeded at a concentration of 2 × 10^6^ cells/mL per well in 24-wells plate (Greiner Bio-One). PBMC were stimulated with an EGFR peptide pool (final concentration 10 μg/mL).

Peripheral blood mononuclear cells cultured with only medium, were used as a negative controls. PBMCs were re-stimulated every 3 days with the EGFR peptide pool and IL-2 (ebiosciences, Birmingham, United Kingdom) (25 UI/mL) until 14 days of stimulation. The EGFR peptide pool was composed by 14 9-mer peptides [(1) ITDFGLAKL; (2) KLFGTSGQK; (3) YLNTVQPTC; (4) TSLGLRSLK; (5) KTIQEVAGY; (6) KVCQGTSNK; (7) MFNNCEVVL; (8) MYYENSYAL; (9) KEITGFLLI; (10) TPPLDPQEL; (11) FLKTIQEVA; (12) VQRNYDDLSF; (13) QFSLAVVSL, and (14) ENNTLVWKY]. Peptides were determined in Base Synthetic Software taking into account those with higher binding for HLA class I, and all peptides were synthesized by the Center of Genetic Engineering and Biotechnology, Havana, Cuba. After incubation, 2 × 10^5^ cells per well in 100 μL was added to the IFN-γ coated ELISPOT. For each patients, triplicate wells were incubated with 100 μL of ConA (sigma), anti-CD3 antibody 1/1000 (Mabtech) (positive control) and 100 μL of the EGFR peptide pool. The plates were incubated for 20–24 h at 37°C in 5% CO_2_. Then, wells were washed with washing buffer and 100 μL of the diluted detection antibody was added to each well. The plates were incubated for 2 h at room temperature. Afterward, plates were washed again and 100 μL of diluted Streptavidine-ALP (1/1000) was added to each well. The plates was incubated for 1 h at room temperature. Finally, the plates were washed once more and 100 μL of substrate solution (BCIP/NBT ALP) was added. Spots parameters were estimated with an ELISpot reader (AID-ELISpot 5.0 software, AID). The results were expressed as percentage of EGFR-specific T cells secreting IFN-γ, after subtracting the number of spots from spontaneous IFN-γ release (unstimulated PBMC) from the number of spots obtained in those wells stimulated with the EGFR peptide pool. Responses were considered positive if 10 or more specific spots were detected and if the number of spots in the presence of an antigen was at least twofold that in its absence.

### Cytokines, Antibodies, and Flow Cytometry

Recombinant (rec) human granulocyte macrophage colony-stimulating factor (GM-CSF), rec. human IL-4, rec. human IL-2, and rec. human IL-7 were purchased from R&D Systems Inc. Anti-EGFR humanized IgG1 mAb nimotuzumab (CIMAHER) was kindly provided by the Center of Molecular Immunology (Havana, Cuba). The EGFR-specific chimeric IgG1 mAb cetuximab (Erbitux; BMS) and the EGFR-specific human IgG2 mAb panitumumab (Vectibix; Amgen) were purchased from the University of Pittsburgh Hillman Cancer Center Pharmacy (Pittsburgh, PA, United States). A human IgG1 isotype control was purchased from Sigma–Aldrich, St Louis, MO, United States. The following fluorophore conjugated antibodies/molecules were used for flow cytometry staining: CD11c FITC, PD-L1 BV421, CD83-PE Cy7, Cy5, CD16-PE-Cy7, EpCAM-APC, TIM3-BV421, PD-1-PerCP/Cy5.5 (clone EH12) were purchased from Biolegend (San Diego, CA, United States); HLA-DR-APC, CD137-PE, CD69-PE-Cy5, CD3-FITC, CD137L-PE, EpCAM-PerCp/Cy5.5, HLA-ABC-PE, CD56 FITC (clone NCAM16.2), CD25-PE-Cy7, CD39 FITC (all BD Biosciences, San Jose, CA, United States); CD56-APC (clone NCAM16.2), CD8-APC, HLA-A^∗^0201-FITC, CD69-PE-Cy7, CD16-PE, CD127-PE, CD4-AF-700, CD3-APC and zombie aqua for cell viability were purchased from BD Pharmingen (San Diego, CA, United States) including their respective isotypes, which were used as negative controls for surface as well as intracellular staining. All mAbs were pre-titrated using activated as well as non-activated PBMC to determine optimal staining dilutions. For flow-cytometric analysis using HLA-A2-peptide tetramers, PE-labeled HLA-A2-EGFR_853-861_ tetramers were obtained from the Tetramer Facility of the NIH (Atlanta, GA, United States). Lymphocytes were prepared for flow cytometry by washing with PBS (Sigma–Aldrich) FACS buffer (2% FBS in PBS). Cells were analyzed on LSRFortessa (Becton Dickinson) using BD FACS DiVa software.

Intracellular Foxp3 staining was performed as described: PBMC were stained with Ab for surface markers and subsequently fixed and permeabilized with TF transcription factor buffer set (BD Bioscience). After washing, cells were subjected to intracellular staining with PE-CF-594 anti-Foxp3 antibody. Flow cytometry of Tregs and NK cells from patients was performed using a Gallios flow cytometer (Beckman Coulter). Data were analyzed using a Kaluza analysis software (Beckman Coulter). The acquisition and analysis gates were restricted to the lymphocyte gate based on characteristic properties of the cells in the forward and side scatter. At least 3 × 10^5^ events were acquired for analysis and gates were restricted to CD3+CD4+ for Tregs and CD3-CD56+ for NK analysis.

### Cytokine Analysis

IFNγ and IL-12 concentrations in the supernatants of activation assays were determined using commercially validated ELISA kit (Invitrogen). A standard calibration curve generated by serial dilutions of recombinant cytokine was used for quantification.

### Statistical Analysis

Statistical significance in EGFR-specific T cells and regulatory T cells and NK cells frequencies were calculated using Wilcoxon matched paired test. T-cell reactivity as measured by the ELISPOT assay was considered positive if 10 or more specific spots were detected and if the number of spots in the presence of an antigen was at least twofold that in its absence. Two-tailed unpaired *t*-test was conducted for statistical analysis to compare the significant difference between two groups. Two-tailed ANOVA was used to compare the significant differences of multiple groups. In all cases *P* < 0.05 was considered significant.

## Results

### Nimotuzumab Induces NK Cell Mediated ADCC of EGFR+ Tumor Targets

**FIGURE 1:**
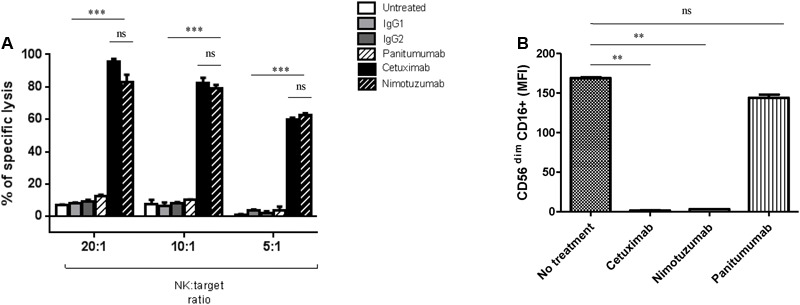
Nimotuzumab induces natural killer (NK) cell-mediated ADCC on EGFR+ tumor cells. **(A)** Isolated NK cells from healthy donors were co-cultured at different ratios E: T 20:1, 10:1, and 5:1 for 4 h with ^51^Cr-labeled JHU029 HNSCC cells coated with panitumumab (10 ug/mL), cetuximab (10 ug/mL) or nimotuzumab (10ug/mL), IgG1 and IgG2 were used as negative controls. Incubation with cetuximab or nimotuzumab demonstrated similar level of specific lysis on tumor cells when compared with either untreated or isotype control (^∗∗∗^*p* < 0.001), while panitumumab did not induce a significant lysis (*p* > 0.05). Graph shows a representative experiment of a triplicate (Two-tailed ANOVA). **(B)** CD16 expression on NK cells was downregulated after nimotuzumab or cetuximab-mediated ADCC (^∗∗^*p* < 0.01). Expression levels of CD16 [represented in mean fluorescence intensity (MFI)] on NK cells co-cultured with PCI-15B (1:1 ratio), conditions were: untreated, cetuximab, nimotuzumab, panitumumab (all at 10 ug/mL, 24 h) were measured. Data are representative of three experiments from 10 different donors. A two-tailed unpaired *t*-test was conducted for statistical analysis.

To investigate if nimotuzumab induces killing of EGFR+ tumor cells, we purified NK cells from healthy donor PBMC by negative magnetic isolation (described in “Materials and Methods”). Purified NK cells were co-cultured with JHU029 cells as target HNSCC cells in the presence or absence of anti-EGFR mAbs (10 ug/mL). ADCC mediated by mAbs were measured by Cr^51^ release. As shown in **Figure 1A**, nimotuzumab induced specific lysis of HNSCC cells at the same level of cetuximab. In contrast, the absence of the mAb or the addition of panitumumab, an IgG2 anti-EGFR mAb which binds poorly to FcγR IIIa on NK cells, were not able to induce NK cell-dependent lysis.

CD16 downmodulation on NK cells has been observed after cetuximab-induced ADCC, supporting the internalization of the FcγR following Fc-FcR binding (Bowles and Weiner, 2005). As expected, nimotuzumab induced significant downregulation of CD16 on NK cells, in contrast to panitumumab and the condition with no mAb (**Figure 1B** and Supplementary Figure 1).

### Nimotuzumab Induces IFNγ Secretion by NK Cells

**FIGURE 2:**
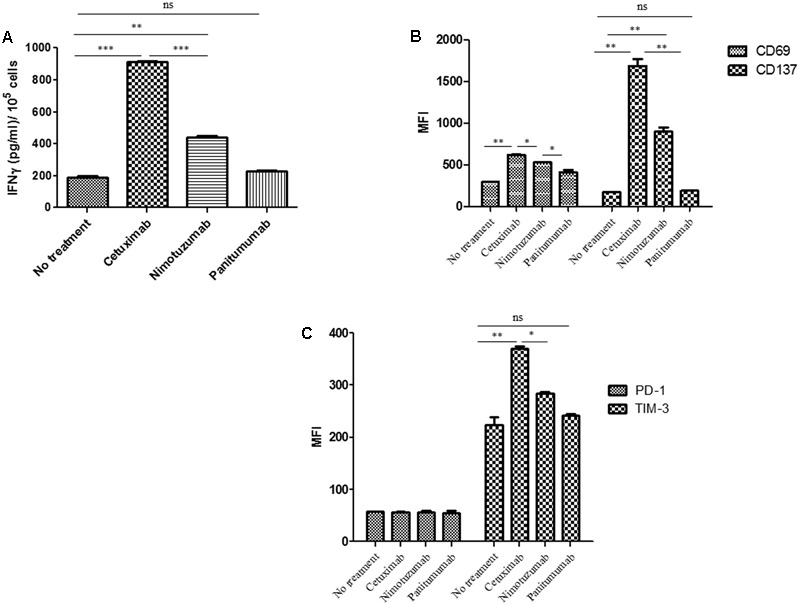
Changes on NK cell marker levels and IFNγ secretion induced by nimotuzumab in the presence of dendritic cells (DCs) and EGFR+ tumor cells. **(A)** The level of IFNγ in the co-culture supernatant of 3 × 10^5^DC: NK: PCI-15B (1:1:1 ratio) with no treatment or with panitumumab or cetuximab or nimotuzumab (each at 10 μg/mL) was measured after 24 h of incubation by ELISA kit. Both cetuximab and nimotuzumab induced IFNγ secretion compared to control. However, the levels induced by nimotuzumab were significantly lower than those induced by cetuximab (^∗∗∗^*p* < 0.001, ^∗∗^*p* < 0.01). Co-cultures without MAbs or with panitumumab don’t induce IFNγ secretion. **(B)** Expression levels (represented in MFI) of activation molecules CD137 and CD69 were analyzed on NK cells co-cultured with DC: PCI-15B (1:1:1 ratio) with no treatment or in the presence of cetuximab or panitumumab or nimotuzumab (each 10 μg/mL, 24 h). Significant lower upregulation of activation markers was obtained for nimotuzumab as compared with cetuximab (^∗∗^*p* < 0.01, ^∗^*p* < 0.05). **(C)** Expression levels (represented in MFI) of inhibitory molecules TIM-3 and PD-1 were analyzed on NK cells co-cultured with DC: PCI-15B (1:1:1 ratio) with no treatment or in the presence of cetuximab or panitumumab or nimotuzumab (each 10 μg/mL, 24 h). TIM-3 was significantly less upregulated when cells were incubated with nimotuzumab as compared with cetuximab (^∗∗^*p* < 0.01, ^∗^*p* < 0.05). No changes were obtained with panitumumab incubation. PD-1 expression levels on NK cells were similar in all conditions. A representative donor out of three is shown. A two tailed unpaired *t*-test was conducted for statistical analysis.

The capacity of cetuximab to increase the secretion of IFNγ by activated NK cells has been previously reported (Lopez-Albaitero et al., 2009a). Notably, DC treated with cetuximab-activated NK cells, further stimulated NK cells in a reciprocal fashion, leading to a significantly increased IFNγ secretion by NK cells. In our experimental design, EGFR + PCI-15B HNSCC cells were incubated with mAbs in the presence of NK cells and autologous DC (**Figure 2A**). Nimotuzumab induced IFNγ secretion by NK cells but it was lower when compared to that of cetuximab. As expected, panitumumab did not induce NK cell activation and IFNγ secretion. The absence of nimotuzumab or cetuximab in the DC: NK: HNSCC co-culture hampered IFNγ secretion as seen in **Figure 2A**.

### Nimotuzumab Enhances NK Cell Activation in the Presence of Autologous DC

To evaluate whether nimotuzumab induces not only IFNγ secretion by NK cells but also the expression of activation markers, we determined the expression level of NK surface markers after co-culture of PCI-15B: NK: DC with nimotuzumab, cetuximab or panitumumab. As depicted in **Figure 2B** and Supplementary Figure 2, NK cells showed significant upregulation of CD137 and CD69 when compared to panitumumab or the untreated condition. However, this upregulation was significantly lower when compared with that of cetuximab. To test if nimotuzumab modified not only activation molecules but also inhibition markers, we analyzed the expression of TIM3 and PD-1 on NK cells under similar conditions. FACS analysis of NK cells showed upregulation of TIM3 but at significant lower level than seen for cetuximab (**Figure 2C** and Supplementary Figure 2). The expression of PD-1 on NK cells was also analyzed since PD-1 is upregulated on NK cells from cancer patients (MacFarlane et al., 2014; Beldi-Ferchiou et al., 2016). The expression of PD-1 on NK cells did not change after HNSCC tumor: NK: DC co-culture regardless the presence of nimotuzumab or cetuximab (**Figure 2C** and Supplementary Figure 2).

### Nimotuzumab Enhances DC Maturation in the Presence of Autologous NK Cells

**FIGURE 3:**
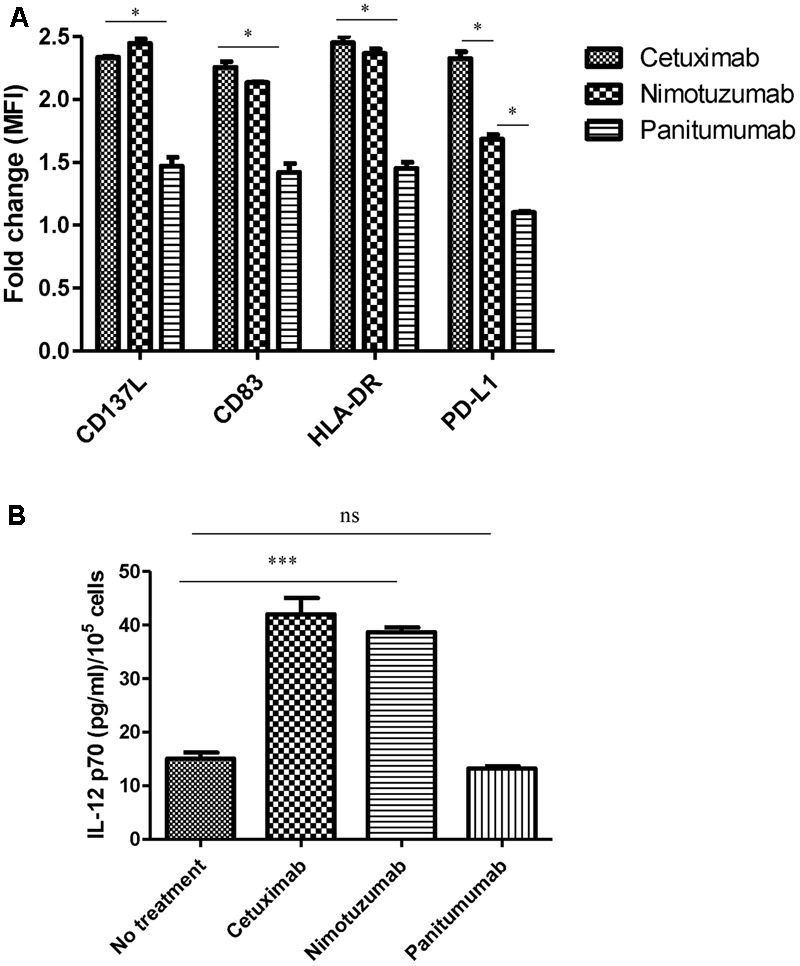
Change on DC marker levels and IL-12 secretion by nimotuzumab-activated NK cells. **(A)** Analysis of CD83/HLA-DR/CD137L/PD-L1 expression on DC co-cultured with NK: PCI-15B with no treatment or in the presence of cetuximab or panitumumab or nimotuzumab (each 10 μg/mL, 48 h). Expression levels of molecules are represented in fold change of MFI related to levels on control DC co-cultured with NK: PCI-15B alone (no treatment). Significant upregulation of CD83/HLA-DR/CD13L were obtained with the use of nimotuzumab or cetuximab in comparison with co-culture without treatment or with panitumumab (^∗^*p* < 0.05). A two-tailed unpaired test was conducted for statistical analysis. Data are representative of three experiments from five different donors. Nimotuzumab induces significant less upregulation of the inhibitory marker PD-L1 on DC as compared with cetuximab (^∗^*p* < 0.05). Data are representative of two experiments from four different donors. **(B)** Enhancement by nimotuzumab of IL-12 secretion in DC-NK co-culture. The level of IL-12 was determine in the supernatant from co-culture of 3 × 10^5^ DC: NK: PCI-15B (1:1:1 ratio) incubated with no treatment or with nimotuzumab or cetuximab or panitumumab (each at 10 mg/mL for 48 h). Values are mean ± SEM of two independent experiments from two separate donors. Similar values were obtained for nimotuzumab and cetuximab while no treatment or panitumumab incubation didn’t induce IL-12 secretion (^∗∗∗^*p* < 0.001). A two-tailed unpaired test was conducted for statistical analysis.

In order to study the capacity of nimotuzumab to induce DC maturation we co-cultured HNSCC tumor cells, NK cells and DCs and determined the expression of surface maturation markers on DC. Despite the lower secretion of IFNγ by nimotuzumab-activated NK cells as compared to cetuximab-treated NK cells, analysis of DC showed significant upregulation of HLA-DR, CD83, and CD137L. The latter molecule is expressed on the surface of antigen presenting cells, including DCs and their precursors. DCs differentiated upon CD137 engagement, are more potent than classical DCs regarding induction of cytotoxic T-cell activity (Ju et al., 2009; Kwajah and Schwarz, 2010). Similar increase was seen on the DC activated by cetuximab-treated NK cell. In contrast, incubation of NK cells with panitumumab, failed to upregulate the expression of some activating molecules such as CD83 and HLA-DR. Other molecule like CD137L was upregulated but at in a lower extent (**Figure 3A** and Supplementary Figure 3).

Activated DCs are able to secrete cytokines such as IL-12, which induces a Th1 phenotype and NK and T cell cytotoxicity. To determine whether nimotuzumab induces secretion of IL-12 in a co-culture system with HNSCC cells: NK: DC, we analyzed the culture supernatants by ELISA. As shown in **Figure 3B**, IL-12 was secreted in a similar concentration when cells were incubated with nimotuzumab or cetuximab. In contrast, panitumumab or media alone failed to induce IL-12 secretion.

### Nimotuzumab and Cetuximab Upregulate Expression of PD-L1 on DC

Since IFNγ is known to be the main inducer of PD-L1 on tumor cells (Mandai et al., 2016) and high amount of IFNγ is released in cetuximab-activated NK cell-treated DC supernatants, we wanted to know if these activated DCs also express inhibitory molecules such as PD-L1. As shown in **Figure 3A** and Supplementary Figure 3, the NK: DC: HNSCC cells co-cultured in the presence of nimotuzumab induced the upregulation of PD-L1 molecule on DCs but was significantly lower as compared with that of cetuximab-treated cells. In contrast, panitumumab did not induce significant PD-L1 upregulation as compared with the baseline (NK: DC: HNSCC tumor cells without mAbs).

### Nimotuzumab Enhances EGFR-Specific CD8+ T Cells in an NK-DC Co-culture System

**FIGURE 4:**
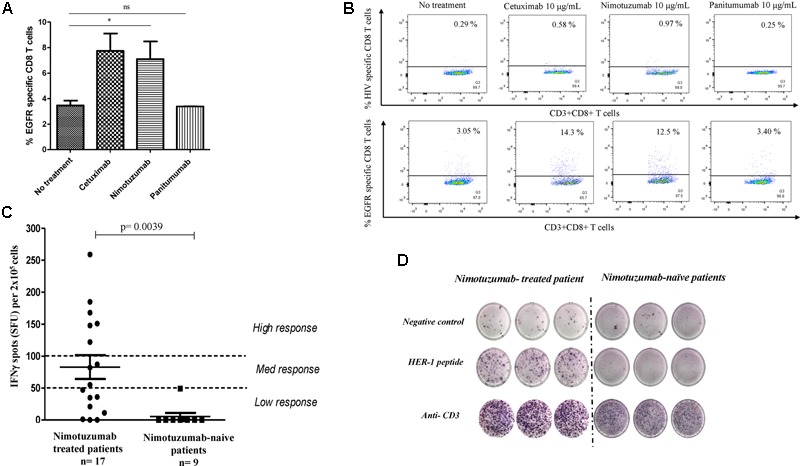
Enhancement by nimotuzumab of IFNγ-secreting EGFR-specific T cells *in vitro* and *in vivo*. **(A)** Purified CD8+ T cells from HLA-A2 healthy donor were stimulated with autologous DC fed with irradiated PCI-15B HNSCC cells (EGFR+ HLA-A2-) coated with nimotuzumab or cetuximab or panitumumab (each at 10 μg/mL) with autologous NK cells (DC:NK:PCI-15B at 1:1:1 ratio). The bar diagram represents the frequency of EGFR_853-861_-specific CD8+ T cells measured by EGFR_853-861_ HLA-A2 tetramer. A significant increase of specific T cells was obtained when the cells were incubated with nimotuzumab or cetuximab (10 μg/mL) in comparison with cells incubated with panitumumab (10 μg/mL) or without treatment (^∗^*p* < 0.05). Data are representative of five individual experiments. A two-tailed unpaired *t*-test was conducted for statistical analysis. **(B)** Dot plot of EGFR-specific CD8+ T cells measured by EGFR_853-861_HLA-A2 tetramer from one representative donor is shown. HIV peptide HLA-A2 tetramer was used as negative control. **(C)** Peripheral blood mononuclear cells (PBMC) were stimulated with a pool of peptides (See “Materials and Methods”) from EGFR and IFNγ-producing T cells in response to stimulation was tested by ELISpot assay. Cells were seeded and analyses performed in triplicates. As negative control no peptide was used. For positive control anti-CD3 mAb was used. Responses were considered positive if 10 or more specific spots were detected and if the number of spots in the presence of an antigen was at least twofold that in its absence. Responses were arbitrarily classified as a low response if the spots were in the range 0–50 spots, medium response if the spots were between 51 and 99 spots and a high response above 100 spots. Higher number of specific T cells in nimotuzumab-treated patients (*n* = 17, 14 HNSCC patients and 3 with other tumor localizations) compared with nimotuzumab-naïve HNSCC patients (*n* = 9) was found. Wilcoxon matched paired test was used as statistical analysis. **(D)** Display result from representative nimotuzumab-treated and nimotuzumab-naive HNSCC patients.

Based on the findings that nimotuzumab was able to induce a cross-talk between DC and NK in the presence of tumor cells, we studied whether these DC cross-presented TAs to specific CD8+ T cells. To assess the *in vitro* cross-priming of T cells by nimotuzumab treatment, we used DCs matured by NK cells that were incubated with nimotuzumab-treated PCI-15B tumor cells. Afterward, the cross-priming of EGFR-specific CTL was measured by flow cytometry using EGFR tetramer. Higher frequency of EGFR-specific CD8+ T cells was found in the case of DC matured with nimotuzumab-activated NK cells and HNSCC cells in comparison with the addition of panitumumab or medium alone. Similar results to nimotuzumab was seen when cetuximab was used as positive control (**Figures 4A,B**).

### Nimotuzumab Increases Frequency of EGFR-Specific T Cell and IFNγ Secretion in Long-Term Treated HNSCC Patients

To evaluate the capacity of nimotuzumab to increase frequency of EGFR-specific T cells *in vivo*, PBMC from long-term nimotuzumab-treated patients (*n* = 17, at least 1 year of nimotuzumab monotherapy) and nimotuzumab-naïve HNSCC patients (*n* = 9) were incubated with a peptide pool from human EGFR. The frequency of IFNγ-secreted T cells was measured by ELISpot assay. A statistically significant higher frequency of IFNγ-secreted T cells specific to EGFR was detected in long-term nimotuzumab-treated HNSCC patients (12 out 14 were considered positive) in comparison with nimotuzumab-naïve patients (**Figure 4C**). ELISPOT of a representative nimotuzumab-treated patient is shown (**Figure 4D**). As expected, a very low frequency of EGFR-specific IFNγ-secreted T cells was found in healthy donors (data not shown).

These data confirm the findings reported for cetuximab, supporting that the treatment with IgG1 anti-EGFR MAb induces the expansion of EGFR-specific T cells in treated HNSCC patients. Whether this expansion is related with a clinical benefit in patients remain to be elucidated.

### T Regs and NK Cells Frequencies in HNSCC Patients Treated with Nimotuzumab

A recent report demonstrated that cetuximab monotherapy increased both circulating and intratumoral CD4+CD39+Foxp3+Tregs which correlated with worse clinical outcome. On the other hand, the frequency of NK cells did not change in PBMC or TIL compartment during cetuximab monotherapy (Mateo et al., 1997; Jie et al., 2015).

**FIGURE 5:**
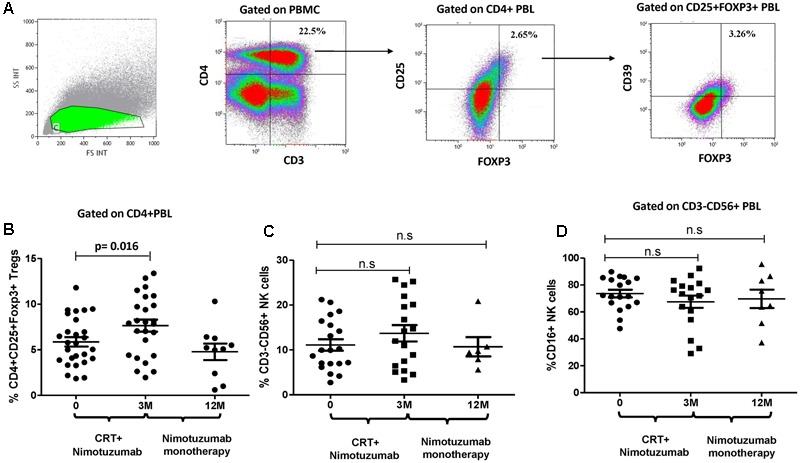
Change on the frequency of circulating CD39+Tregs and CD3-CD56+ NK cells during nimotuzumab treatment. **(A)** Gate analysis of circulating CD25^hi^CD39+Foxp3+Tregs in CD4+PBMC isolated from one patient is shown. **(B)** Percentage of circulating CD25^hi^CD39+Foxp3+Tregs from 29 HNSCC patients were performed at baseline, after chemoradiation (CRT) with nimotuzumab (M3, *n* = 25) and at the end of nimotuzumab monotherapy (M12, *n* = 9). Combination of chemoradiation and nimotuzumab increase Tregs frequency as compared to baseline values (*p* = 0.016, Wilcoxon matched paired test). Percentage of Tregs decreases to baseline numbers at the end of nimotuzumab treatment. **(C)** The frequency of CD56+ NK cells and cytotoxic population CD16+ NK cells **(D)** was analyzed in CD3-PBMC from HNSCC patients at baseline (D0, *n* = 21), after chemoradiation combined with nimotuzumab (M3, *n* = 19) and at the end of nimotuzumab monotherapy (M12, *n* = 8). Non-significant differences in NK cell levels were found (*p* > 0.05, Wilcoxon matched paired test).

To investigate the effects of nimotuzumab on circulating Treg and NK cells of HNSCC patients, the frequency of CD4+CD25+CD39+Foxp3+Treg and CD56+CD16+ NK cells in PBMC was analyzed before and after therapy. Cells measurements were performed prior nimotuzumab and CRT and compared with post-treatment frequencies measured after induction phase (nimotuzumab and CRT) and at the end of nimotuzumab maintenance therapy. As shown in **Figures 5A,B**, the frequency of CD4+CD25+CD39+Foxp3+Treg significantly increased in these HNSCC patients after induction phase (3 months) as compared to baseline Treg levels. This effect was reversed after nimotuzumab monotherapy. Interestingly, the frequency of CD4+CD25+CD39+Foxp3+Treg decreased to baseline values at the end of maintenance phase with nimotuzumab monotherapy. In contrast, the percentage of total NK cells and CD16+ NK cells did not change during therapy, which is in line with what was found for cetuximab (**Figures 5C,D**).

## Discussion

The role of innate and adaptive immune response in the clinical efficacy of TA-targeted mAb has been suggested based mainly on the preclinical results (Abes et al., 2010; Park et al., 2010). Recently, data about TA specific T cell response found in cancer patients treated with cetuximab has been previously reported (Srivastava et al., 2013; Jie et al., 2015). Nimotuzumab is a humanized mAb for the treatment of EGFR over-expressing tumors and has been used with success in advanced unresectable locoregional HNSCC patients (Basavaraj et al., 2010; Reddy et al., 2014). The survival advantage and long-term duration of effect noticed after few weeks of treatment with nimotuzumab, suggested that inhibition of EGFR signal transduction and tumor proliferation are not the only effector mechanism involved. In the preclinical studies, it was demonstrated that nimotuzumab is a strong antitumor drug both for *in vitro* and for *in vivo* setting (Crombet-Ramos et al., 2002). However, the capacity of nimotuzumab to kill EGFR + tumor cells by other effector mechanisms and to induce an innate and adaptive immune response had not been studied so far. Here we showed, for the first time, that nimotuzumab can induce NK cell-mediated ADCC at similar levels than cetuximab, despite the lower affinity of nimotuzumab for the EGFR (Garrido et al., 2011). ADCC is elicited by ligation of a mAb-coated specific target cells and FcγR IIIa (CD16) on NK cells, which starts a sequence of events ending in the secretion of IFNγ and granzyme-containing granules (Roda et al., 2006; Lopez-Albaitero et al., 2009a). It is known that ADCC activity depends on different factors such as IgG isotype, Fc glycosylation and EGFR expression on target cells (Patel et al., 2010). On one hand, nimotuzumab and cetuximab are IgG1 mAb, isotype which binds with high affinity to human FcR I and FcR IIIa. This antibody isotype can efficiently mediate a strong *in vitro* ADCC response, while marginal ADCC is observed with the IgG_2_ mAb such as panitumumab. Previous studies have established that cetuximab can induce ADCC *in vitro*, even in tumor cells with relatively low levels of EGFR expression (Kawaguchi et al., 2007; Kurai et al., 2007). However, direct correlation between EGFR expression and ADCC activity has been found for nimotuzumab (manuscript in preparation). Since it has been reported that a threshold antigen density on target cells is required to mount an effective ADCC response (Velders et al., 1998; Niwa et al., 2005), it may be that the HNSCC cell line used in this study express the EGFR density enough for nimotuzumab to elicit high ADCC activity. Another important issue is the CD16 polymorphisms which determine the bond strength to antibody F_C_ portion (Musolino et al., 2008; Bibeau et al., 2009). In the case of nimotuzumab this factor remains to be studied. CD16 downmodulation on NK cells has been observed after cetuximab-induced ADCC supporting the internalization of the FcγR following Fc-FcR binding (Bowles and Weiner, 2005). As it was expected, for both cetuximab and nimotuzumab a CD16 downmodulation was detected after ADCC while no change in CD16 expression was observed for panitumumab treatment.

Crosslinking of the Fc portion with CD16 activates not only NK cytotoxicity but also upregulates expression of the co-stimulatory receptor CD137 (4-1BB), CD69 and production of IFNγ (Clausen et al., 2003; Lin et al., 2008). Afterward activation, CD137 is expressed on different immune cells (Vinay and Kwon, 2014). Remarkably, NK cells upregulate the expression of CD137 when they face a mAb bound to tumor cells. Previous publications reported that cover of tumor cells with either rituximab or trastuzumab increases the CD137 expression on NK cells (Kohrt et al., 2011, 2012). It was recently published that cetuximab upregulates the CD137 expression on human NK cells after *in vitro* incubation with tumor cells expressing EGFR (Kohrt et al., 2014). This is in line with our results which show a significant upregulation of CD137 on NK cells from healthy donors in the presence of cetuximab, tumor cells, and DC. Interestingly, upregulation of CD137 on NK cells is induced after their exposure to nimotuzumab-coated tumor cells but the surface expression was lower as compared with that of cetuximab. In a recent publication, upregulation of CD137 in intratumoral NK cells was found in neoadjuvant cetuximab-treated HNSCC patients. It was correlated with FcγR IIIa V/F polymorphism and predicted clinical response (Srivastava et al., 2016). *In vivo* upregulation of CD137 remains to be measured in further clinical trials using nimotuzumab.

Another cell surface molecule present on activated NK cells that triggers their spontaneous cytotoxicity is the early activation marker CD69 (Borrego et al., 1999). An increase in CD69 expression is accompanied by an enhanced cytotoxicity against various target cells (Lanier et al., 1988). In recent studies, the authors showed that increased expression of NKG2D and CD69, and downregulation of killing inhibitory receptor (KIR) was seen when NK cells were spread in CD16 antibody-coated flasks. Cytokine detection indicated that these NK cells increased IFN-γ secretion (Niu et al., 2015). As it happened for CD137 expression, the upregulation of CD69 on NK cells was significantly lower when nimotuzumab-bound tumor cells was used in comparison with cetuximab-activated NK cells. Additionally, nimotuzumab-activated NK cells secreted significantly higher IFNγ amount as compared to cells incubated with panitumumab or without treatment but the secretion level was significantly lower than that of cetuximab incubation. All these findings suggest that in spite of the same level of cytotoxicity (measured by ADCC) induced by nimotuzumab and cetuximab, less NK cell activation is induced by nimotuzumab. The capacity to induce ADCC has been measured by others anti-EGFR mAb (Ferris et al., 2010) but the activation of NK cells during this process has not been studied. For the first time, we published the NK cell activation by another IgG1 anti-EGFR mAb. Since nimotuzumab and cetuximab exhibit the same IgG1 isotype and similar glycosylation status, the cause of the difference in IFNγ secretion and expression of activation molecules may be in the lower affinity of nimotuzumab by EGFR which is considered intermediate (KD ∼10^-8^ M for the Fab fragment), and constitutes a 10-fold lower affinity in comparison with the cetuximab Fab fragment (Li et al., 2005). As it was previously reported and in contrast with cetuximab, nimotuzumab required bivalent binding for maintaining stability with the EGFR in the cell surface, conducted to nimotuzumab preferentially binding to cells that present moderate to high EGFR expression levels (Garrido et al., 2011). However, if EGFR density is low, nimotuzumab monovalent binding is transient, while cetuximab keeps interacting strongly with the receptors. Although both HNSCC lines used for ADCC and NK cell activation experiments exhibited high EGFR expression, it is higher in the one used for cytotoxicity (JHU029) as compared with the line selected for activation assays (PCI-15B) (personal communication). This finding might explain the same ADCC levels obtained for both mAb and the differences in NK cell activation.

In addition to activation molecules both cetuximab and nimotuzumab-activated NK cells increase the TIM-3 expression, although in the case of nimotuzumab the upregulation of TIM-3 on NK cells was significantly lower as compared with cetuximab. TIM-3 molecule plays a critical role in immunoregulation (Sabatos et al., 2003). In cancer, the expression of TIM-3 on T lymphocytes may promote T cell exhaustion and the expansion of suppressive CD4+FoxP3+ regulatory T cells and CD11b+Gr-1+ myeloid-derived suppressor cells (MDSC) (Han et al., 2013; Sakuishi et al., 2013). Some authors have found that stimulation of NK cells that express TIM-3 induces cytokine secretion and cytotoxic activity (Ndhlovu et al., 2012). In a different study, the presence of galectin-9, a TIM-3 ligand, significantly increased NK cell-derived IFN-γ production (Gleason et al., 2012). Recent papers, however, published contradictory results concerning the role of TIM-3 on NK cells (Ju et al., 2010; da Silva et al., 2014). HBV patients showed high expression of TIM-3 on NK cells, and blockade of TIM-3 enhanced cytotoxic activity and IFN-γ secretion *in vitro* (Ju et al., 2010). TIM-3 is also upregulated on NK cells from cancer patients with poor prognosis (Baksh and Weber, 2015). In this sense, it was reported increased upregulation of TIM-3 on NK cells which showed an exhausted phenotype in advanced melanoma patients (da Silva et al., 2014). All these findings suggest that TIM-3 signaling seems to have a suppressive function for NK cell effector activities. Interestingly, the studies showing TIM-3 as an NK cell activation marker focused on healthy donors, while those involving TIM-3 as an NK cell suppression molecule studied patients with chronic diseases. That is why this distinction may be crucial for understanding the flexible function of TIM-3 depending on the context.

The expression of TIM-3 on NK cells during the ADCC-induced by cetuximab has not been published before. According to our findings we can speculate that cetuximab-mediated ADCC activates NK cells and upregulates expression of costimulatory molecules and IFNγ secretion. At the same time, inhibitory molecules may be upregulated inducing NK cell exhaustion. In contrast, ADCC-induced by nimotuzumab may induce less NK cell activation and less exhaustion. More experimentation should be performed in order to test this hypothesis.

We confirmed that cetuximab-activated NK cells induced IFNγ-dependent DC maturation. The co-activation of cetuximab-activated NK cells and autologous DC likely promotes a DC-1 polarization with IL-12 secretion and Th1 cytokines. Despite the lower levels of IFNγ detected after nimotuzumab-activated NK cells, autologous DC were fully matured as indicated by the concomitant and similar upregulation of the costimulatory molecules CD83, CD137L, and HLA-DR on the DC surface. Additionally, similar levels of IL-12 were detected in cell culture supernatant regardless of the incubation with nimotuzumab or cetuximab. Both nimotuzumab and cetuximab EGFR+ tumor complex in the presence of NK and DC generate cross-presentation of TAs by DC to T cells resulting in the induction of EGFR-specific T cells *in vitro*. As expected, panitumumab was not able to induce neither NK-DC cell crosstalk nor TA specific T cell generation. Interestingly, in the maturation process of DC as a consequence of cetuximab-activated NK cells, PD-L1 was upregulated on DC surface. PD-L1 upregulation was significantly lower in the presence of nimotuzumab. As it is known PD-1 on T cells and PD-L1 on DC participate in the maintenance of peripheral tolerance by decreasing T cell-DC interactions (Fife et al., 2009). Upon stimulation with pro-inflammatory cytokines like IFNγ, PD-L1 is induced not only on APC but also in tumors. This negative regulatory loop may decrease the T cell and NK cell activity in tumor microenvironment, which in our case might be more evident in the case of cetuximab which induces both higher IFNγ secretion and PD-L1 expression by immune cells.

Our *in vitro* data showed that nimotuzumab-mediated ADCC, activated NK cells which induced IFNγ-dependent DC maturation, enhancing antigen presentation and cross-priming of EGFR-specific CD8+ T lymphocytes. Similar results have been shown for other mAbs anti-EGFR such as: cetuximab using HNSCC cells and trastuzumab in breast carcinoma bearing-mice (Wolpoe et al., 2003; Srivastava et al., 2013). Although the detection of TA specific T cells *in vivo* has been a rare finding in patients treated with mAbs, it was recently identified circulating EGFR-specific T cells in cetuximab-treated patients with HNSCC (Srivastava et al., 2013). In this case, T cell response was restricted to HLA-A2.1 peptides, since only patients with this HLA restriction were studied. Similar results were obtained for long-term nimotuzumab-treated HNSCC patients in which higher IFNγ-producing T cells specific to EGFR peptide pool (including different HLA restrictions) were found in the PBMC of HNSCC as compared with non-treated HNSCC patients. Our study reports, for the first time, the presence of circulating TA specific T cells in HNSCC patients with at least 1 year treatment with nimotuzumab. Whether this biomarker is a surrogate of clinical benefit remains to be confirmed in larger clinical trials.

Although nimotuzumab induces tumor cell death through NK ADCC, the frequency of peripheral CD16+NK cell remains constant during nimotuzumab-based treatment. This finding is in line with the results previously reported for cetuximab. In the case of cetuximab, circulating NK cells showed increased cytotoxic molecules after antibody treatment (Jie et al., 2015). In order to validate the role of NK cells in nimotuzumab mechanism of action, future clinical trials should characterize the NK cells phenotype and function not only in periphery but also in the tumor site of treated patients.

It was recently reported that cetuximab-based monotherapy is associated with increased circulating and intratumoral Tregs in treated HNSCC patients. These Tregs were able to suppress NK cell-mediated ADCC. Moreover, higher levels of Tregs correlated with worse prognosis in cetuximab-treated patients (Jie et al., 2015). In contrast, patients treated with nimotuzumab monotherapy showed a decrease in Tregs frequency as compared to the end of chemoradiation combined with nimotuzumab period. The ability of cisplatin-based chemotherapy and radiation to decrease the numbers of CD4+T cells but to increase the percentage of CD4+CD39+ Tregs in head and neck cancer patients has been previously published. The levels of these highly suppressive Tregs remained elevated for long-term period (Schuler et al., 2013). In our case, we found the highest frequency of Tregs after the end of combined treatment likely due to the resistance of Tregs to chemoradiation. Interestingly, although it was not statistically significant, a decrease in circulating Treg frequency was observed at the end of nimotuzumab monotherapy. This result suggests that chronic use of nimotuzumab doesn’t promote Treg expansion, at least, in the periphery. This finding is in contrast to what was published for cetuximab which showed increased Treg frequency impairing cetuximab-mediated ADCC activity (Jie et al., 2015). Nevertheless, the infiltration of Tregs at the tumor site and the correlation with clinical outcome should be addressed in further clinical trials with nimotuzumab.

In summary we report, for the first time, the capacity of the anti-EGFR mAb nimotuzumab to induce ADCC-mediated tumor cell killing and adaptive immunity through TA specific T cells. In light of these findings the study of immune-infiltration at the tumor site and its correlation with clinical outcome of treated patients should be addressed in further clinical trials.

## Author Contributions

Conception and design: ZM, AL, FC-B, RS, IC, CF, BFM, MB, TC, and RF. Development of methodology: ZM, AL, FC-B., AV, RS, TG-B, and MB. Acquisition of data (provided animals, acquired and managed patients, provided facilities, etc.): ZM, AL, FC-B, EH, ZG, AG, ML, IC, CF, BFM, and MB. Immunological assessments: ZM, AL, FC-B, and AV. Analysis and interpretation of data (e.g., statistical analysis, biostatistics, computacional analysis): ZM, AL, FC-B, AV, RS, TG-B, and RF. Writing, review, and/or revision of the manuscript: ZM, AL, FC-B, TG-B, TC, and RF. Administrative, technical, or material support (i.e., reporting or organizing data, constructing databases): TC and RF. Study supervision: TC and RF. Final approval: TC and RF.

## Conflict of Interest Statement

RF: consulting or advisory role: AstraZeneca/Medimmune, Bristol Myers-Squibb, Merck. Research funding: AstraZeneca/Medimmune (Inst.), Bristol-Myers Squibb (Inst.), and VentiRx (Inst.). The other authors declare that the research was conducted in the absence of any commercial or financial relationships that could be construed as a potential conflict of interest.
