# Supplementary material for: Nimotuzumab Induces NK Cell Activation, Cytotoxicity, Dendritic Cell Maturation and Expansion of EGFR-Specific T Cells in Head and Neck Cancer Patients
Source: Front Pharmacol. 2017 Jun 19;8:382. doi: 10.3389/fphar.2017.00382 (PMC5474456; doi:10.3389/fphar.2017.00382)
Supplement: Supplementary file 1 [file Presentation_1.PDF]

Supplementary Material

Nimotuzumab induces NK cell activation, cytotoxicity, dendritic cell maturation and expansion of EGFR-specific T cells in head and neck cancer patients

Mazorra Z.[1§*], Lavastida A.[1§], Concha-Benavente F.[2], Valdés A.[1], Srivastava R.M.[3], García-Bates T. M.[3,4], Hechavarría E.[1], González Z.[1], González A.[1], Lugiollo M.[5], Cuevas I.[5], Frómeta C.[5], Fernández B.[5], Barroso M.C.[6], Crombet T.[6] and Ferris R.L.[2,3,7]

Correspondence to: Dr. Zaima Mazorra. Clinical Immunology Department, Center of Molecular Immunology; 216 St and 15 Ave., Atabey, Playa, Havana 11600, Cuba. Phone: 53-7-2713114, Fax: 53-7-2720644; E-mail: zaima@cim.sld.cu

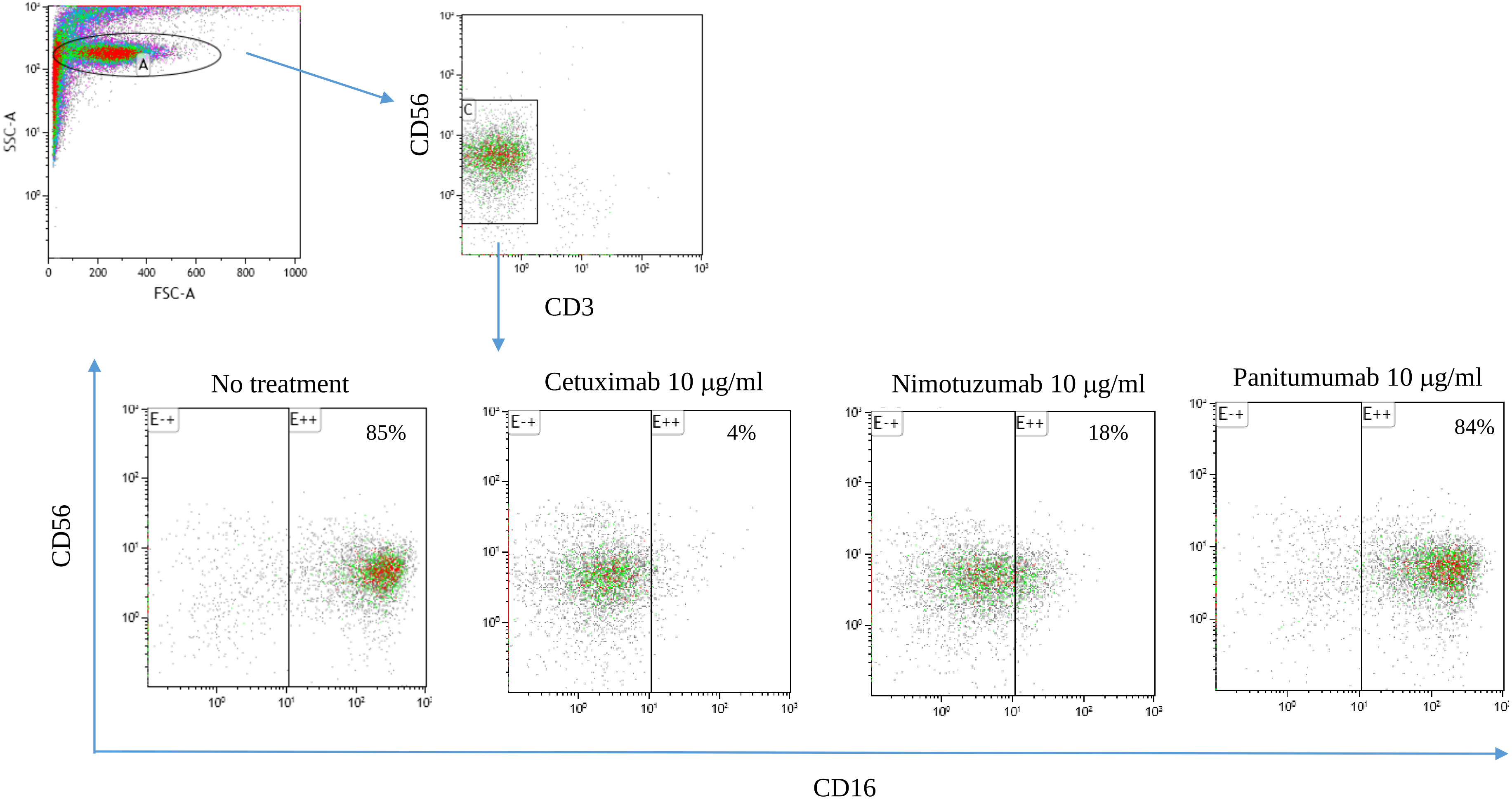

Supplementary Figure 1: NK cells downmodulate CD16 expression after cetuximab or nimotuzumab –mediated ADCC. NK cells were co-cultured with PCI-15B (1:1 ratio) with no treatment or cetuximab or nimotuzumab or panitumumab (each 10 μg/mL, 24h). Dot plots show the percentages of CD16+ NK cells from a representative experiment.

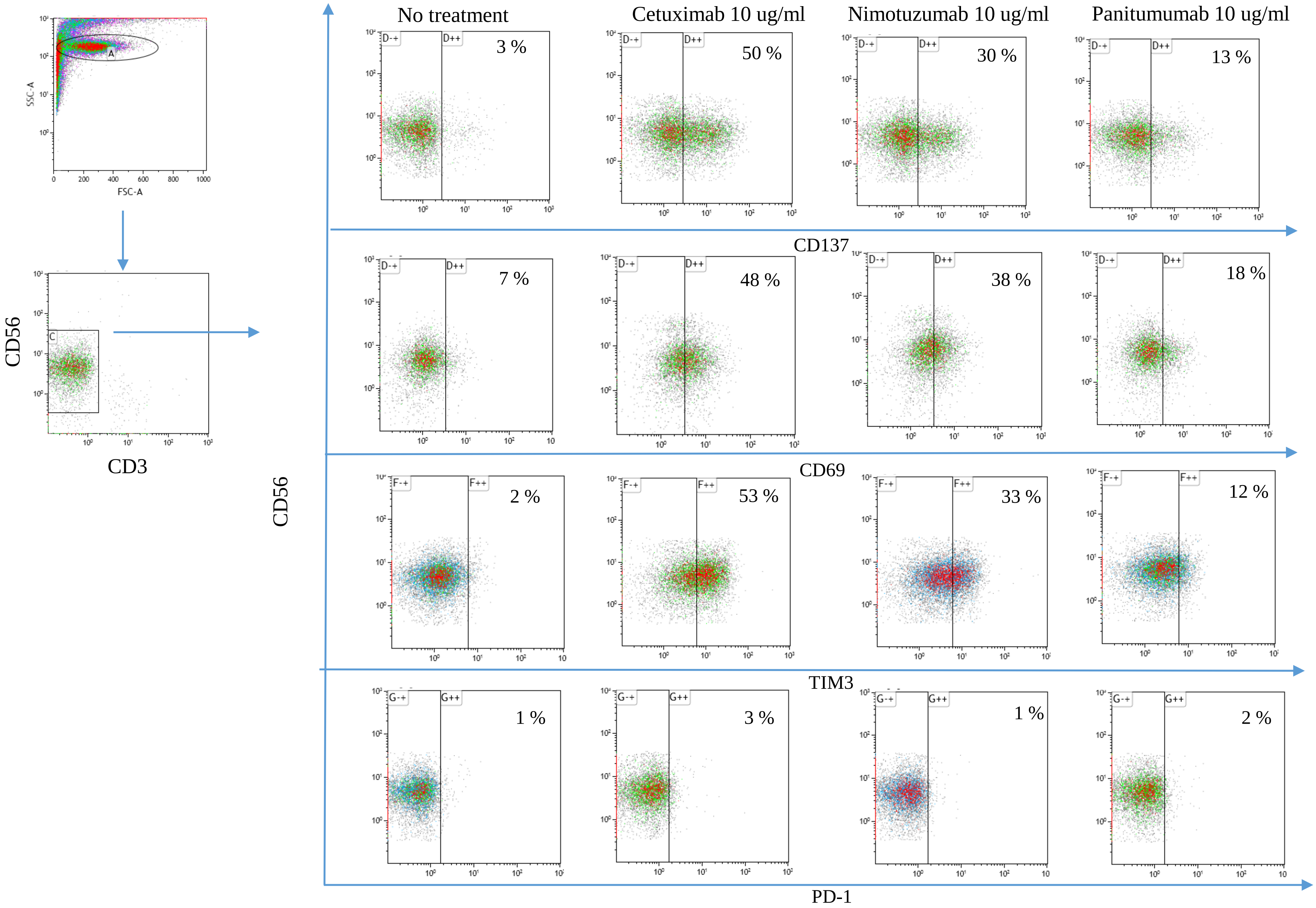

Supplementary Figure 2: Change on activation and inhibition markers expression on NK cells is induced by nimotuzumab in the presence of DC and EGFR + tumor cells. NK cells were cocultured with DC: PCI-15B (1:1:1 ratio) in the presence of cetuximab or nimotuzumab or panitumumab (each 10 μg/mL) or without treatment during 24 h. Dot plots show the percentages of activated NK cells (CD137 and CD69), TIM3+ and PD-1+ NK cells from a representative experiment.

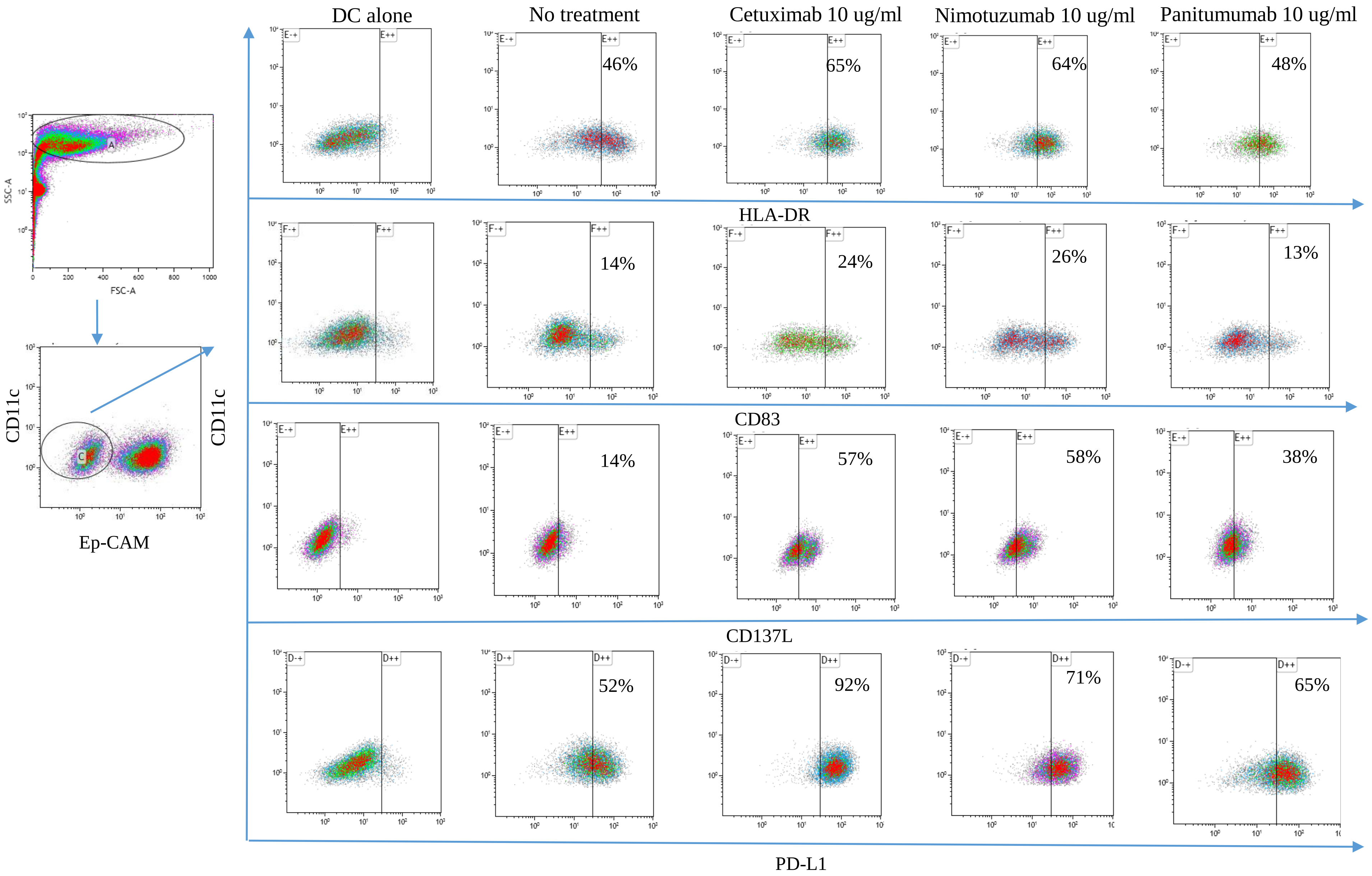

Supplementary Figure 3: Change on DC markers expression is induced by nimotuzumab- activated NK cells. DCs were co-cultured with NK: PCI-15B (1:1:1 ratio) in the presence of cetuximab or nimotuzumab or panitumumab (each 10 µg/mL) or without treatment during 48 h. Dot plots show the percentages of maturated DC (HLA-DR, CD83, and CD137L) and PD-L1+DC from a representative experiment. Expression levels of markers on DCs alone are displayed
